# An activator for pyruvoyl-dependent l-aspartate *α*-decarboxylase is conserved in a small group of the γ-proteobacteria including *Escherichia coli*

**DOI:** 10.1002/mbo3.34

**Published:** 2012-08-14

**Authors:** Shingo Nozaki, Michael E Webb, Hironori Niki

**Affiliations:** 1 Microbial Genetics Laboratory, Genetic Strains Research Center, National Institute of Genetics1111 Yata, Mishima, Shizuoka, 411-8540, Japan; 2 Astbury Centre for Structural Molecular Biology and School of Chemistry, University of LeedsLeeds, LS2 9JT, UK; 3 Department of Genetics, Graduate University for Advanced Studies (Sokendai)1111 Yata, Mishima, Shizuoka, 411-8540, Japan

**Keywords:** ADC, β-alanine, coenzyme A, PanD, pantothenate, YhhK

## Abstract

In bacteria, *β*-alanine is formed via the action of l-aspartate *α*-decarboxylase (PanD) which is one of the small class of pyruvoyl-dependent enzymes. The pyruvoyl cofactor in these enzymes is formed via the intramolecular rearrangement of a serine residue in the peptide backbone leading to chain cleavage and formation of the covalently-bound cofactor from the serine residue. This reaction was previously thought to be uncatalysed. Here we show that in *Escherichia coli*, PanD is activated by the putative acetyltransferase YhhK, subsequently termed PanZ. Activation of PanD both in vivo and in vitro is PanZ-dependent. PanZ binds to PanD, and we demonstrate that a PanZ(N45A) site-directed mutant is unable to enhance cleavage of the proenzyme PanD despite retaining affinity for PanD. This suggests that the putative acetyltransferases domain of PanZ may be responsible for activation to enhance the processing of PanD. Although *panD* is conserved among most bacteria, the *panZ* gene is conserved only in *E. coli-*related enterobacterial species including *Shigella, Salmonella*, *Klebsiella* and *Yersinia*. These bacteria are found predominantly in the gut flora where pantothenate is abundant and regulation of PanD by PanZ allows these organisms to closely regulate production of *β*-alanine and hence pantothenate in response to metabolic demand.

## Introduction

**Figure 1:**
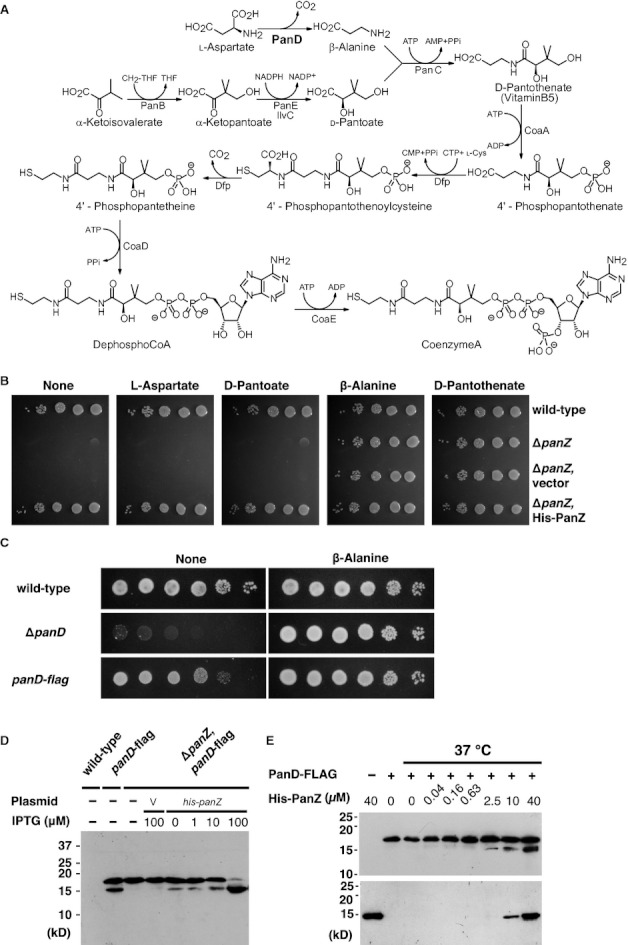
Supplements for growth defects of the Δ*yhhK* mutant and in vivo and in vitro assay for cleavage of *panD*. (A) Biosynthetic pathway of pantothenate and coenzyme A. (B) Growth of the Δ*panZ* (Δ*yhhK*) mutant on agar plates of the M9 glucose medium with or without supplements is indicated above each panel. Cell culture overnight was applied at a series of dilutions of 1:10. These cells were spotted on the plates and incubated at 37°C for 2 days. Wild-type; MG1655, Δ*panZ*; SN202, Δ*panZ*/vector; SN202 harboring pCA24N(-GFP), Δ*panZ/*His-*panZ*; SN202 harboring pCA24N(-GFP)-*his-panZ*. (C) Growth of the *panD-flag* strain on agar plates of the M9 glucose medium with or without *β*-alanine. The Δ*panD* strain was used as negative control. Wild-type; MG1655, ΔPanZ; SN202, ΔPanZ-*flag*; SN208. (D) In vivo cleavage of *panD*-FLAG was analyzed by SDS polyacrylamide gel electrophoresis (SDS-PAGE) and detected by immunoblot analysis using anti FLAG antibody. Wild type; MG1655, *panD-flag*; SN208, Δ*panZ, panD-flag*; SN216. pCA24N(-GFP) was transformed into SN216 (V), and pCA24N(-GFP)-*his-panZ* expressing His-PanZ are designated *his-PanZ*. Expression of his-PanZ was induced by IPTG. (E) In vitro cleavage of purified *panD*-FLAG was analyzed by SDS-PAGE and detected by immunoblot analysis using anti FLAG (Upper) or anti His antibody (Lower). Purified His-PanZ (40 μmol/L) and PanD-FLAG (2.5 μmol/L) are shown in lane 1, 2, respectively. Purified PanD-FLAG (2.5 μmol/L) was incubated at 37°C for 3 h with or without His-PanZ.

Pantothenate (vitamin B5) is required in all organisms for the synthesis of coenzyme A (CoA) and acyl carrier proteins, however its biosynthesis is limited to prokaryotes, fungi and plants in which it is produced from D-pantoate and *β*-alanine (Webb et al. 2004; Fig. 1A). In bacteria, *β*-alanine is synthesized via decarboxylation of l-aspartate, a reaction catalyzed by the pyruvoyl-dependent enzyme l-aspartate *α*-decarboxylase (ADC). ADC-dependent synthesis of *β*-alanine is the sole source of *β*-alanine and deficiency in ADC *β*-alanine therefore leads to *β*-alanine auxotrophy which can be relieved with either *β*-alanine or pantothenate. In *Escherichia coli*, supplementation of culture media with pantothenate or *β*-alanine markedly increases the cellular level of CoA, whereas supplementation with excess pantoate is much less effective (Cronan et al. 1982). This demonstrates that *β*-alanine supply is limiting for pantothenate biosynthesis in this organism. Regulation of ADC activity is therefore central to the regulation of intracellular pantothenate and CoA concentrations.

The enzyme is a member of the small class of pyruvoyl-dependent enzymes, which contain a covalently-bound pyruvoyl cofactor (Williamson and Brown 1979). This enzyme class includes human *S*-adenosyl methionine decarboxylase, human phosphatidylserine decarboxylase and histidine decarboxylase from *Lactobacillus* 30a (van Poelje and Snell 1990). For all of these enzymes, the protein is initially translated as an inactive proenzyme (designated the π-protein) subsequently is activated via post-translational modification. A serine residue in the peptide backbone rearranges to generate an ester intermediate (Albert et al. 1998) which is subsequently cleaved to generate the *α*-subunit and *β*-subunit observed in the active enzyme. Cleavage of the peptide chain generates a dehydroalanyl residue at the N-terminus of the *α*-subunit which hydrolyses to generate the pyruvoyl group (van Poelje and Snell 1990; Schmitzberger et al. 2003). In all cases, cleavage of the π-chain is autocatalytic; the π-chain of purified *E. coli* PanD is activated to generate the *α*-subunit and the *β*-subunit of ADC by auto-activation in vitro (Ramjee et al. 1997). Auto-activation of purified *E. coli* PanD is however slow; although activation occurs to some extent after incubation at 37°C for 24 h, stoichiometric activation requires higher temperatures or longer incubation times. Considering the doubling time of *E. coli* cells, which is about 20 min at 37°C under optimal conditions, this rate for activation of PanD to generate ADC is relatively slow compared with in vivo. Naturally, it was therefore proposed that there might be a specific catalyst for proenzyme processing in vivo (Ramjee et al. 1997). Activation of pyruvoyl-dependent S-adenosylmethionine decarboxylase is stimulated by addition of putrescine and spermidine, these molecules react with the product of S-AdometDC, and thereby can regulate its production. More recently it has been found that cleavage of histidine decarboxylase, is activated by a protein activator, HdcB, which acts enzymatically in maturation (Trip et al. 2011). However, no such activator has been identified for ADC.

Reinvestigation of the genetic analysis of mutants requiring pantothenate suggested the existence of an activator in *Salmonella typhimurium* and *E. coli*. Mutations generating *β*-alanine auxotrophy were identified in the 1970s and 1980s. These mutations were mapped to two different chromosomal positions at 5 min and 89 min in *Salmonella typhimurium* (Ortega et al. 1975; Primerano and Burns 1983). The gene encoding ADC, *PanD,* was mapped at 5 min in *S. typhimurium* and genetically linked to *panB* and *panC*, which encode the two other essential enzymes for pantothenate biosynthesis (Primerano and Burns 1983). In *E. coli*, PanD was mapped at 3 min and was closely linked to *panC* (Cronan 1980) corresponding, therefore, to the mutation in *S. typhimurium* at 5 min. However, the second mutation at 89 min was forgotten for decades without further study, during which time it had been thought that the metabolic pathway of pantothenate was completely elucidated in bacteria (Webb et al. 2004). Here we report that this “forgotten mutation” is required for activation of ADC.

## Results

### Requirement of β-alanine for pantothenate synthesis in the *yhhK* gene deletion mutant

A gene-knockout library of *E. coli* strains revealed a number of gene knock-out mutants which, while essential for growth of *E. coli* in minimal synthetic media (Baba et al. 2006; Joyce et al. 2006), are dispensable in nutrient-rich media. This media-dependent growth inhibition suggests that these knock-out mutations cause nutritional deficiencies. However, in most cases, the biological functions of these genes remain unknown. One such gene knock-out mutant is Δ*yhhK*. Supplementation of growth media with pantothenate restored the growth of Δ*yhhK* cells even in synthetic minimal media (Adams et al. 1990); it seemed likely therefore that Δ*yhhK* cells are deficient in the biosynthetic pathway for pantothenate. We examined which intermediate during pantothenate synthesis was required for normal growth (Fig. 1B). Pantothenate is generated via the condensation of *β*-alanine and pantoate. Supplementation of the growth medium with *β*-alanine, but not with pantoate, restored the growth of Δ*yhhK* cells, whereas supplementation with l-aspartate, the precursor of *β*-alanine, did not. Complementation of Δ*yhhK* cells, using a plasmid encoding a his-tagged YhhK, efficiently restored the normal growth of Δ*yhhK* cells. We therefore concluded that biosynthesis of *β*-alanine is prevented by the knock-out mutation of *yhhK*. In bacteria, *β*-alanine is formed by decarboxylation of l-aspartate. This enzymatic reaction is catalyzed by l-aspartate-*α*-decarboxylase (ADC), encoded by PanD (Webb et al. 2004). In *E. coli*, the locus of *yhhK* does not correspond to the locus of PanD. In *S. typhimurium*, two loci for *β*-alanine auxotrophy have been identified, one corresponding to PanD and the other located at 89 min. The genetic configuration of *E. coli* and *S. typhimurium* chromosomes resemble each other, and so we theorized that the map position of the latter mutant in *S. typhimurium* might correspond to that of *yhhK* in *E. coli*, which was indeed the case. We will therefore henceforth refer to *yhhK* as *PanZ*.

### Activation of cleavage of PanD by PanZ (YhhK) for the maturation of ADC

How does the PanZ gene product influence the decarboxylation of l-aspartate to generate *β*-alanine? We hypothesized that the conversion of PanD to an active enzyme could be accelerated by PanZ. To assess whether PanZ is an activator for the maturation of ADC, we examined the cleavage of PanD in ΔPanZ cells. To detect cleavage of PanD in vivo, FLAG peptides were added to the C-terminus of PanD (PanD-FLAG). The growth rate of the cells with PanD-FLAG was slower on minimal synthetic medium as compared to wild type cells, but they did not require *β*-alanine as a supplementary nutrient (Fig. 1C) demonstrating that PanD-FLAG is a functional enzyme. In PanZ^+^ cells, two bands at 18 kDa and 15 kDa corresponding to the unactivated PanD gene product and the *α*-chain of the activated ADC were detected by Western blotting analysis (Figure 1D). The 15 kDa band was however not observed in extracts of ΔPanZ cells. Complementation of the ΔPanZ cells with his-tagged PanZ (*his*-PanZ) led to detection of the 15 kDa band. High-level induction of His-PanZ using 100 μmol/L of isopropyl *β*-d-1-thiogalactopyranoside (IPTG) led to near disappearance of the 18 kDa band corresponding to the inactive PanD and the presence of only the 15 kDa band corresponding to the activated protein. These results indicate that PanZ stimulates cleavage of the PanD zymogen in cells and that it is essential for this reaction. We next determined whether PanZ was sufficient for the activation of PanD to form ADC by in vitro analysis of cleavage of purified PanD-FLAG by His-PanZ (Fig. 1E). After incubation for 3 h at 37°C, PanD-FLAG on its own is unactivated (Fig. 1E). However, addition of His-panz leads to complete activation of PanD-FLAG. On the timescale shown (3 h) however, addition of 16 equivalents of His-PanZ is insufficient to fully activate PanD-FLAG. Thus, while PanD is cleaved in a PanZ-dependent manner in vivo and in vitro, it appears that this process is not catalytic.

### Involvement of the GNAT domain in PanD cleavage

**Figure 2:**
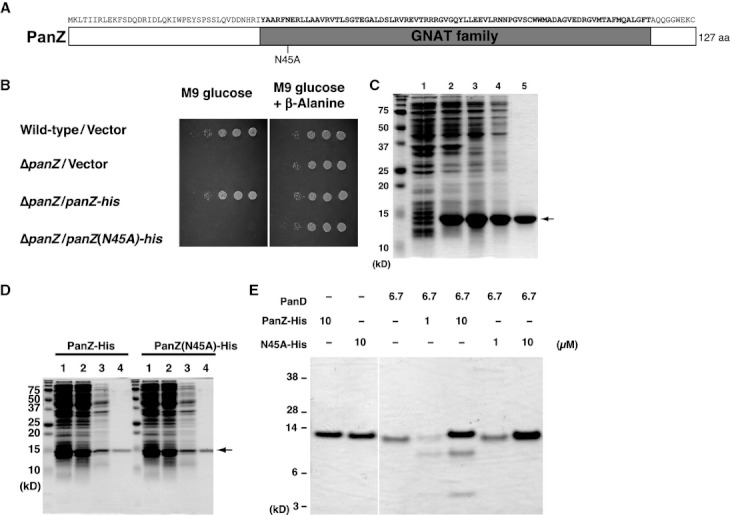
Effect of a single mutation in the GNAT domain on cell growth and in vitro cleavage of *panD*. (A) Amino acid sequence and a schematic structure of PanZ with the position of the point mutation in GNAT family domain. (B) Complementation of phenotype of Δ*panZ* by *panZ(N45A)-his*. Growth of Δ*panZ* derivative strains on agar plates of M9 glucose medium with or without *β*-Alanine. Wild-type; MG1655/pBAD24, Δ*panZ*; SN202/pBAD24, Δ*panZ*/vector; SN202/pBAD24-*panZ*-*his*, Δ*panZ/panZ*-*his*; SN202/pBAD24-*panZ*-*his*, Δ*panZ/panZ*(N45A)-*his*; SN202/pBAD24-*panZ*(N45A)-*his*. (C) Purification of PanD. Lane 1; total cell lysate, lane2; total cell lysate after addition of IPTG, lane3; soluble fraction of cell lysate, lane4; fraction of 40-50% ammonium sulphate precipitation, lane5; fraction of anion-exchange column purification. (D) Purification of PanZ-His and PanZ(N45A)-His. Lane1; total cell lysate after addition of IPTG, lane2; soluble fraction, lane3; fraction of 50–80% ammonium sulphate precipitation, lane4; fraction of Ni-sepharose column purification. (E) In vitro cleavage of purified PanD. Purified proteins were incubated at 37°C for 60 min in 50 mmol/L HEPES/KOH, 100 mmol/L potassium acetate, 1 mmol/L EDTA, pH 7.6 and subjected to SDS-PAGE and CBB staining.

Bioinformatics analysis showed that PanZ is a putative N-acetyltransferase and a member of the GNAT (Gcn5-related N-acetyltransferase) superfamily of acetyltransferases (Fig 2 A). Indeed, the structure of PanZ (YhhK) bound to CoA has been solved using nuclear magnetic resonance (NMR, PDB ID: 2k5t). We used ITC to confirm the interaction between PanZ and CoA, and also acetyl CoA (data not shown). Several residues in the acetyltransferase domain are well conserved in the GNAT superfamily between bacteria and humans. Residue Asn45 is one such residue, and it is completely conserved in those bacterial species which have the PanZ gene. We constructed a single mutation in PanZ at one of these positions to generate a N45A site-directed mutant, this mutant PanZ(N45A) could not compensate for the requirement for *β*-alanine in ΔPanZ cells (Fig 2B).

To confirm that this was due to a deficiency in the catalytic activity of PanZ(N45A), we tested cleavage of PanD using purified proteins. Native PanD was purified from cell lysates that overproduced PanD (Fig 2C). In contrast, PanZ and PanZ(N45A) proteins were purified using his-tags and affinity purification (Fig 2D). After incubation of a mixture including purified PanD and PanZ-His, cleaved fragments of PanD were detected by SDS gel electrophoresis (Fig. 2E). This cleavage reaction was independent of CoA or acetyl-CoA, and no change in the cleavage reaction could be detected by the addition of either (data not shown). However, the cleavage reaction could not be detected when PanZ(N45A)-His was used in place of PanZ-His; the single amino acid substitution in the GNAT domain prevents PanZ from activating PanD. Hence, the GNAT domain is responsible for maturation of PanD to ADC.

### *yhhK* is conserved only among γ-proteobacteria

**Figure 3:**
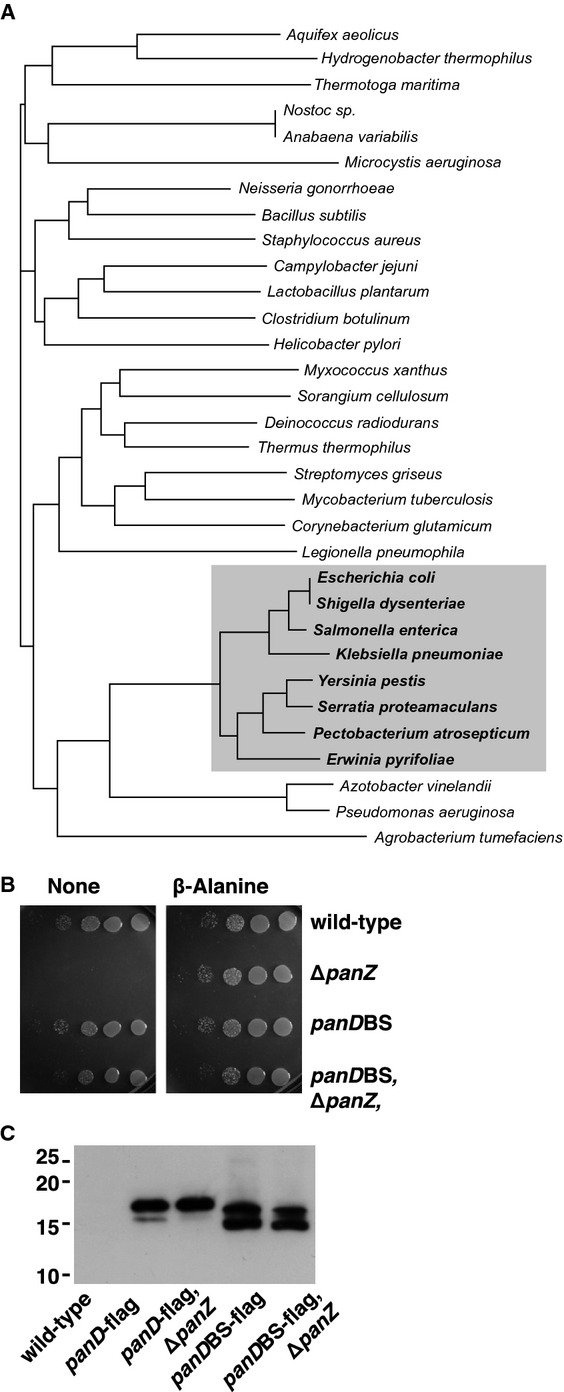
Evolutionary relationships in the *panD* and *panZ* families. (A) Phylogenetic tree of *panD* in representative bacterial species. A gray box indicates that homologues of the *panZ* (*yhKK*) gene are conserved (B) Growth of the *E. coli* cells with *panD*_*BS*_ was tested on agar plates of the M9 glucose medium with or without *β*-alanine. Overnight cell culture was spotted at a series of dilutions of 1:10. wild type; MG1655, Δ*panZ*; SN202, *panD*_*BS*_; SN219, *panD*_*BS*_*,* Δ*panZ*; SN223. (C) In vivo cleavage of PanD_BS_-FLAG was analyzed by SDS-PAGE and detected by western blotting using anti FLAG antibody. Wild type; MG1655, *panD-flag*; SN208, Δ*panZ, panD-flag*;SN216, *panD*_*BS*_*-flag*; SN220, Δ*panZ, panD*_*BS*_*-flag*;SN224.

Orthologous genes encoding ADC are conserved among almost all bacterial species, whereas homologous genes of PanZ are found only in some members of the γ-proteobacteria including *E. coli* (Fig. 3A).

The observation of PanD in bacterial species lacking PanZ homologues suggests that other activating factors might be involved in the activation of PanD or that, in these organisms, auto-activation of PanD alone might produce active ADC. We tested whether PanD derived from *Bacillus subtilis*, which lacks a PanZ homologue, could suppress the *β*-alanine auxotrophy of *E. coli* ΔPanZ cells. The cloned PanD gene of *B. subtilis* (PanD_*BS*_) was substituted for PanD on the *E. coli* chromosome. *E. coli PanZ*^+^ cells in which PanD is replaced with PanD_*BS*_ grew as well as wild type *E. coli* in M9 glucose medium (Fig. 3B). The same substitution in *E. coli ΔPanZ* generates cells that can also grow without pantothenate supplementation. These results suggest that activation of PanD_BS_ does not require PanZ. We confirmed the cleavage of PanD_BS_ in *E. coli*. A C-terminal FLAG tag was added to the chromosomal PanD_*BS*_ and cell extracts were monitored by western blotting. Two bands at ∼17 kDa (corresponding to the PanD_BS_ proenzyme) and ∼14 kDa (the activated *α*-subunit) were observed in both *pan*Z^+^ and ΔPanZ cells (Fig. 3C). Although the proenzyme form of PanD_BS_-FLAG was observed in the cell extract, almost all of the proenzyme form was completely changed to the cleaved form during purification (data not shown) preventing study of the activation of PanD_BS_ in vitro.

### Physical interaction of PanD with PanZ

**Figure 4:**
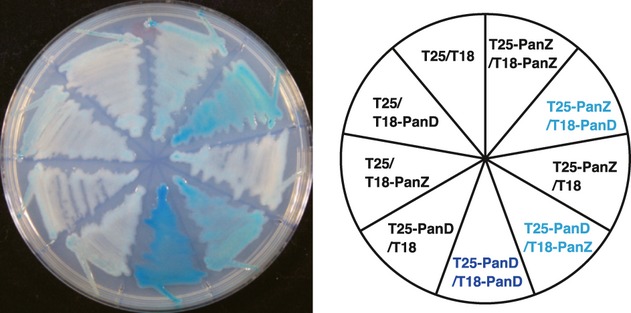
Bacterial two hybrid assay. The combinations of the prey and the bait are indicate in the right panel.

Acceleration of PanD activation by PanZ suggests that non-covalent interaction of the proteins may be required. We examined the protein-protein interaction between PanZ and PanD in vivo using a bacterial two-hybrid assay (Karimova et al. 1998). Each of PanZ and PanD were fused to fragments of the catalytic domain of adenylate cyclase (T25 or T180). Association between PanZ and PanD leads to association of the fragments of adenylate cyclase, further leading to cAMP production which subsequently induces expression of *lacZ*. Homotetramerization of PanD was used as a positive control (Ramjee et al. 1997; Albert et al. 1998); cells that co-expressed T25-PanD and T18-PanD fusions appeared dark blue when grown on X-gal media (Fig. 4). This intense color reflects the strong interactions within the homotetramer - boiling in the presence of SDS is required to fully dissociate the PanD tetramer (Ramjee et al. 1997). Co-expression of either the T25-PanD and the T18-PanZ fusion, or the T18-PanD and the T25-PanZ fusion led to generation of a pale blue color (Fig. 4A) indicating an interaction between PanZ and PanD (though weaker than the very strong PanD tetramerization). Finally, co-expression of T25-PanZ and T18-PanZ leads to a very pale blue color suggesting multimerisation of PanZ is occurring, although however this could be a PanD tetramer mediated interaction.

**Figure 5:**
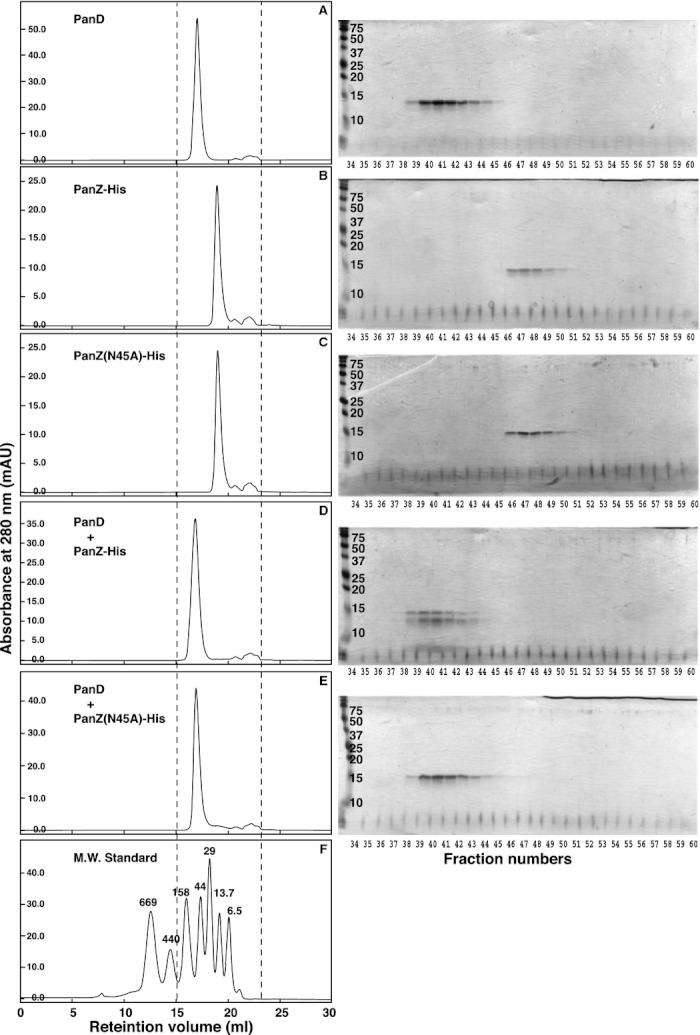
Gel filtration chromatography for analysis of affinity between PanD and PanZ. Purified proteins were chromatographed by gel filtration and elution profiles were monitored by absorbance at 280 nm. (A) PanD. (B) PanZ-His. (C) PanZ(N45A)-His. (D) PanD and PanZ-His. (E) PanD and PanZ(N45A)-His. (F) Molecular weight standards. Molecular weights are indicated in the elution curve (kDa). Fractions between the broken line (from fraction number 34 to 60) were subjected to SDS-PAGE and CBB staining as shown in the right panels. Left lane in each panel indicates molecular weight (kDa).

Physical interaction between PanZ and PanD in vitro was then confirmed by gel filtration with purified PanZ and PanD. Although PanZ-His and PanD have similar molecular weights, 15.6 kDa and 13.8 kDa respectively, retention time of the purified protein, PanZ-His and PanD are distinctive (Fig. 5A and B). Rather, the retention time of PanD corresponds to a tetramer. After co-incubation of PanZ-His and PanD, both proteins co-eluted together with an earlier retention time (Fig. 5D). Analysis by gel electrophoresis indicated that PanD had been cleaved and the PanZ-His was co-purified with the cleaved PanD. This suggests that PanZ tightly binds to the PanD tetramer even after maturation of the inactive proenzyme. In order to confirm the affinity of PanZ(N45A)-His for the unactivated proenzyme form of PanD, the purified PanZ(N45A)-His was also eluted using gel filtration chromatography (Fig. 5C and E). PanZ(N45A)-His co-purified with PanD, suggesting that the mutant PanZ still retains the ability to bind to PanD. However, the processing of the unactivated proenzyme form of PanD was completely inhibited. Thus, the mutated residue is critical for cleavage of the proenzyme rather than perturbing its affinity for the substrate. Addition of CoA or acetyl-CoA did not affect the elution of PanD, PanZ and the mixture as before (data not shown).

## Discussion

The putative acetyltransferase PanZ is crucial for the activation of the proenzyme PanD in vivo. This function of PanZ appears to not be catalytic, but rather to provide a cofactor to stimulate self-activation of PanD. Although PanZ can bind to CoA and acetyl-CoA, we could not detect any influence of the addition of CoA and acetyl-CoA to the in vitro reaction. PanZ with the single mutation of N45A demonstrates that the well conserved feature of this domain between the GNAT family domain, which can bind CoA, is involved in enhancing the self-processing of PanD. This suggests that binding of a CoA derivative to PanZ might regulatactivation of the processing of proenzyme PanD in vivo. It is possible that a CoA derivative has been co-purified with PanZ and is therefore present in our experiments to investigate the interaction between PanZ and PanD. More precise biochemical measurements are therefore needed to observe the influence of CoA and acetyl-CoA on the in vitro reaction.

**Figure 6:**
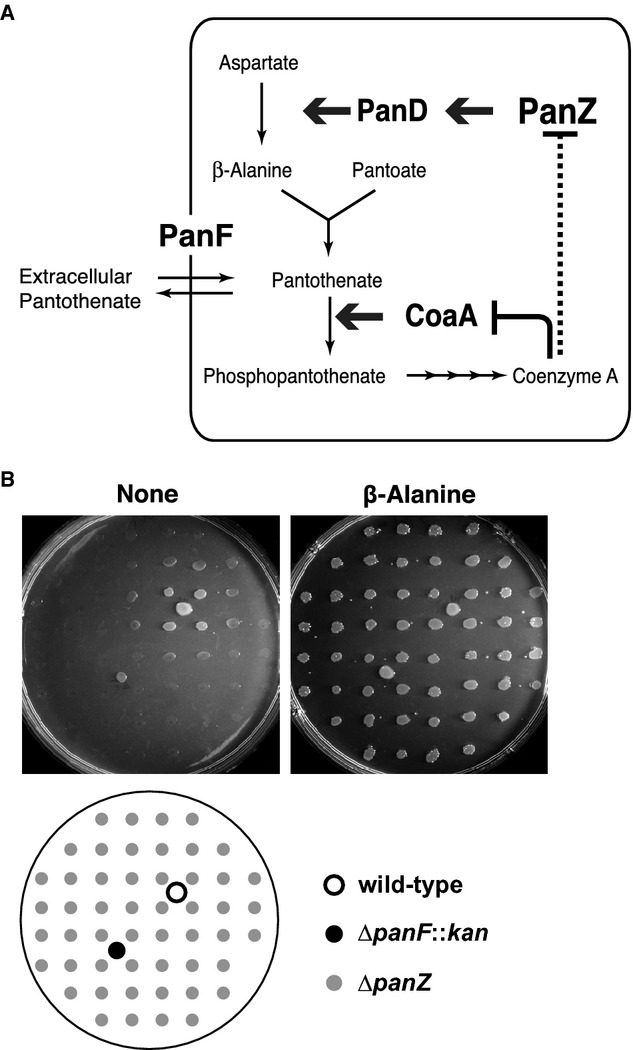
A model of feedback regulation of pantothenate synthesis by CoA and effect of PanF on cell growth. (A) Regulation of pantothenate synthesis and its cellular content in *E. coli*. Pantothenate is produced by condensation of *β*-alanine and pantonate. The following phosphorylation of pantothenate by CoaA is negatively regulated by CoA. After inhibition of the metabolic step of CoA synthesis, pantothenate is still produced in the cells and then an excess of pantothenate is secreted toward the living environment by PanF, which is a sodium dependent transporter. PanF can also incorporate extracellular pantothenate. Activation of PanD is dependent on PanZ. We speculate that CoA binds to PanZ when activation of PanD might be down. This secondary feed back loop allow cells to stop the pantothenate synthesis, in addition to CoaA. (B) The phenotype of the *panZ* mutant is characterized by a requirement of *β*-alanine that suppressed by neighbors of the wild type cells in a *panF* dependent manner. Cells were grown on the M9 medium with or without *β*-alanine and incubated at 37°C for 2 days. Open, closed and grey circles indicate the spot of wild-type cells, Δ*panF*::*kan* cells and Δ*panZ* cells, respectively.

Is it possible and physiologically significant that somehow CoA or acetyl CoA binding regulate the activation of PanD in the generation of ADC? As CoA is derived from pantothenate, we would therefore expect CoA to negatively regulate the synthesis of pantothenate. Such a regulatory pathway would provide a second negative feedback in the pantothenate synthetic pathway. In most organisms except archaea, pantothenate kinase, CoaA, phosphorylates pantothenate to produce phosphopantothenate (Brown 1959) (Fig. 6A). CoaA activity is regulated by CoA in a negative feedback manner (Vallari and Rock 1987; Vallari and Jackowski 1988). Inhibition of CoaA leads to accumulation of pantothenate and the excess pantothenate is secreted from the cells via the sodium-dependent transporter PanF (Jackowski and Rock 1981; Vallari and Rock 1985). This means that ΔPanZ cells can grow by using pantothenate secreted by wild type cells (Fig. 6B). Indeed, slight growth of ΔPanZ cells was often seen in proximity to growing PanZ^+^ cells. When the ΔPanZ cells were inoculated near pantothenate transport-deficient cells (Δ*panF*) however, no growth was observed (Fig. 6B). Thus, secreted pantothenate can be used by those bacteria that cannot produce or otherwise do not produce pantothenate.

In the gut flora, vast numbers of microorganisms live and produce pantothenate. However feedback control of CoA on CoaA activity does not effectively regulate this pathway. The production of pantoate from *α*-ketoisovaleric acid via ketopantoate is essentially reversible. ATP-dependent synthesis of pantothenate from pantoate and *β*-alanine therefore represents the first committed step in the biosynthetic pathway. In *E. coli*, the flux through this step is limited by the supply of *β*-alanine, formed by decarboxylation of l-aspartate. Control of *β*-alanine supply via control of ADC activation would therefore prevent depletion of l-aspartate and intermediates in branched chain amino acid biosynthesis.

As PanZ is a putative acetyltransferase, we therefore hypothesize that acetylation of PanD may regulate PanD activation in response to the ratio of cellular acetyl-CoA and CoA. Both small molecules bind tightly to PanZ. Therefore, if acetylation inhibits formation of ADC then acetylation will only occur when acetyl-CoA accumulates in the cell. Such accumulation could be a signal to repress pantothenate biosynthesis, despite the presence of numerous potential acetylation sites in PanD, we have not been able to obtain evidence of acetylation suggesting that a more complex mechanism is required for regulation of PanD activation in vivo.

In contrast to PanD from *E. coli*, PanD_BS_ does not require PanZ for its activation. Overexpression of PanD from *Corynebacterium glutamicum* in *E. coli* has previously been reported as a high yielding strategy for production of *β*-alanine and hence pantothenate (Dusch et al. 1999). This is, perhaps, consistent with our findings: an analogue of the PanZ gene is not present in *C. glutamicum* and so, like PanD_BS_, PanD from *C. glutamicum* might be fully activated by auto-processing. Previous attempts to engineer the pathway in plants by Fouad et al. (Fouad and Rathinasabapathi 2006)and Chakauya et al. (Chakauya et al. 2008) have used PanD_EC_. As PanD_EC_ requires PanZ for its activation, PanD_BS_ would therefore be more effective in these applications. It remains unclear which structural differences between these orthologs lead to rapid activation of PanD_BS_ without the addition of PanZ. Alignment of orthologs of PanD (Fig. S1) reveals that the N-terminal (1-111) portions of the proteins are highly conserved among all bacterial species. However, the last 20 aa are poorly conserved among most bacterial species, but are well conserved in those bacterial species in which orthologs of PanZ are found. This region is distant from the site of cleavage (Gly24-Ser25) so it is not clear how this region could prevent activation of PanD in these species. Recently, it has been reported that this portion of the protein can bind intermolecularly to the active site of adjacent proteins as a result of crystal packing (Webb et al. 2012); it is therefore possible that such intramolecular interactions might act to inhibit activation in solution. We are currently investigating the role of this and other semi-conserved residues in the activation of PanD.

In summary, we have identified an essential new component in the pantothenate biosynthetic pathway. The pathway of pantothenate biosynthesis has been previously proposed as a target for antimicrobial chemotherapy (Spry et al. 2008). But as the pathway is common to all bacteria, the opportunity for selective chemotherapy is limited. PanZ homologues are however only found in the genomes of *E. coli* and related bacteria, such as *Shigella, Salmonella, Klebsiella* and *Yersinia*, all of which are resident in the animal gut. PanZ therefore provides a potential target by which to inhibit only pantothenate biosynthesis in these pathogenic bacteria, while sparing the gut flora where pantothenate is abundant.

## Experimental Procedures

### Bacterial strains and plasmids

Table 1 shows bacterial strains and plasmids used in this report. To construct the *PanD-flag* gene of SN208, we used a DNA fragment encoding the FLAG octapeptide repeated three times in tandem (3× *flag*) with the *cat* gene inserted between *frt* sites (*frt-cat-frt*). The *frt* sites are recognition sequences of the sequence specific recombinase FLP. The DNA fragment (3× *flag-frt-cat-frt*) was amplified by polymerase chain reaction (PCR) using primers PanDflagU and PanDflagL. We used high-fidelity KOD-plus DNA polymerase (TOYOBO, Osaka) for PCR. The resulting fragment was used for transformation into MG1655 according to the Datsenko and Wanner's procedure (Datsenko and Wanner 2000). Then the 3× *flag* tag was thereby fused with the carboxyl terminus of the PanD gene. If necessary, the *cat* genetic marker was removed using the FLP-FRP recombination (Datsenko and Wanner 2000).

**Table 1:** Strains and plasmids

Strain or plasmid	Parental strains, genotype and description	
MG1655	Wild-type *E. coli*	
SN202	MG1655 Δ*panZ*	This study
SN205	MG1655 Δ*panD*	This study
SN208	MG1655 *panD-flag*	This study
SN216	MG1655 *panD-flag* Δ*panZ*	This study
SN219	MG1655 Δ*panD*::*panD*_*BS*_	This study
SN223	MG1655 Δ*panD*::*panD*_*BS*_ Δ*panZ*	This study
SN220	MG1655 *panD*_*BS*_*-flag*	This study
SN224	MG1655 *panD*_*BS*_*-flag* Δ*panZ*	This study
SN225	MG1655 Δ*panF*::*kan*	This study
SN227	BL21(DE3) Δ*panZ*	This study
DHM1	*cya*	Karimova et al.
pET28Ap-*panD*	*panD* expression plasmid	This study
pBAD24-*panD-flag*	*panD*-FLAG expression plasmid	This study
pBAD24-*panZ*-*his*	*panZ*-His expression plasmid	This study
pBAD24-*panZ* (N45A) -*his*	*panZ*(N45A)-His expression plasmid	This study
pCA24N(-GFP)	ASKA clone vector without *gfp*	Kitagawa et al.
pCA24N(-GFP)-*his-panZ*	6xHis-*panZ* expression plasmid	Kitagawa et al.
pKT25	Bacterial two-hybrid vector	Karimova et al.
pUT18C	Bacterial two-hybrid vector	Karimova et al.
pUT18C-*panZ*	T18-*panZ* expression plasmid	This study
pUT18C-*panD*	T18-*panD* expression plasmid	This study
pKT25-*panZ*	T25-*panZ* expression plasmid	This study
pKT25-*panD*	T25-*panD* expression plasmid	This study

To construct the PanD_*BS*_ strain (SN219), the PanD gene of *B. subtilis* 168 was amplified using BSPD1 and BSPD21 as PCR primers. The *frt-cat-frt* fragment was also amplified from template DNA of *PanD-flag-frt-cat-frt* using primers of BSPD31 and PanDflagL. As the sequence of BSPD21 is complementary to the sequence BSPD31, the amplified DNA fragment encoding PanD of *B. subtilis* could anneal with the *PanD-flag-frt-cat-frt* DNA fragment to, yielding the PanD_*BS*_*-frt-cat-frt* DNA fragment. This DNA fragment was used to substitute *E. coli PanD* with PanD_*BS*_ by recombination according to Datsenko and Wanner's procedure (Datsenko and Wanner 2000). Similarly a strain with PanD_*BS*_*-flag* (SN220) was constructed using primers BSPD1 and BSPD4 for amplification of PanD_*BS*_, and primers of BSPD5 and PanDflagL for that of *flag-frt-cat-frt*.

To construct Δ*yhhK* (PanZ) derivatives, Δ*yhhK*::*kan*, which is one of the deletion mutants in the Keio collection (Baba et al. 2006), was used to transduce each parental strain by P1 transduction, resulting in SN202, SN216, SN223 and SN224. The *kan* gene marker was eliminated according to Datsenko and Wanner's procedure (Datsenko and Wanner 2000). To construct the Δ*panF* strain (SN225), Δ*panF*::*kan* of the KEIO collection was introduced to MG1655 by P1 transduction.

pCA24N(-GFP)-*his-PanZ* is one of the expression plasmids of the ASKA clone (-) library, in which each cloned gene can be expressed by addition of IPTG (isopropyl-*β*-d-thiogalactopyranoside) with the gene product having an N-terminal His tag (Kitagawa et al. 2005).

To construct pBAD24-*PanD-flag*, the *PanD-flag* fragment was amplified by PCR using the primers ECPanDBADU and PanDL. The PCR product was digested with EcoRI and SphI and inserted into the EcoRI-SphI site of the pBAD24 vector plasmid (Guzman et al. 1995). Expression of the *PanD-flag* gene is induced by addition of arabinose. However, leaky, uninduced expression could complement the phenotype of ΔPanD. The vector pBAD24-*PanZ-his* was constructed similarly except that the PanZ gene was amplified using the primers PanZBADU and PanZBADL.

To construct pBAD24-PanZ(N45A)-*his*, a single nucleotide mutation was introduced into the PCR primers PanZN45AL and PanZN45AU. The 5' portion of the PanZ-*his* gene was amplified using the primers of PanZBADU and PanZN45AL, and the 3' portion using primers of PanZN45AU and PanZBADL. As the primer sequence of PanZN45AL is complementary to that of PanZN45AU, the PCR products could be annealed and thus the mutated PanZ gene was obtained. This PanZ (N45A)-*his* fragment was cloned into the pBAD24 vector as described above.

To construct an expression plasmid for PanZ, the PanZ gene was amplified using primers THPanZU and THPanZL. The PCR product was digested with EcoRI and BamHI and inserted into the EcoRI-BamHI site of pUT18C and pKT25 (Karimova et al. 1998), respectively, resulting in pUT18C-PanZ and pKT25-PanZ. pUT18C-PanD and pKT25-PanD were constructed similarly except that the PanD gene was amplified by primers THPanDU and THPanDL.

We converted the gene selection marker of pET28 (Novagen), which is an IPTG-inducible expression vector, from a kanamycin resistance gene to an ampicillin resistance gene. To construct pET28Ap, pET28 plasmid DNA was digested with AlwNI and ClaI to eliminate the kanamycin resistant gene. pBR322 plasmid DNA was digested with AlwNI and ClaI to obtain the ampicillin resistance gene, and the DNA fragment was ligated into the above pET28 plasmid DNA. To construct pET28Ap-PanD, the PanD gene was amplified from MG1655genomic DNA using the primers PanDPETU and PanDPETL. The PCR product was digested with NcoI and HindIII and introduced into the NcoI-HindII site of pET28Ap.

Strains of the KEIO collection were obtained from the National BioResource Project of the National Institute of Genetics, Mishima, Japan.

### Purification of His-PanZ

ΔPanZ cells harboring pCA24N(-GFP)-*his-PanZ* were incubated in 1000 ml of L broth containing 10 μg/ml of chloramphenicol at 30°C until OD_600_ reached 0.5, and then isopropyl-*β*-d-thiogalactopyranoside (IPTG) was added to a final concentration of 1mmol/L in order to express His-PanZ protein. The culture was incubated for an additional 4 h. Cells were harvested by centrifugation and resuspended in 10 ml of Lysis Buffer (50 mmol/L NaH_2_PO_4_, 300 mmol/L NaCl, 10 mmol/L imidazole 1 mg/ml lysozyme, pH 8.0) and incubated on ice for 30 min. Cells were disrupted by sonication and cell debris was removed by centrifugation to obtain a cleared lysate. Two ml of 50% slurry of Ni-NTA agarose (QIAGEN) was added to the cleared lysate and mixed by shaking at 4°C for 1 h. The lysate-Ni-NTA mixture was loaded into a column and washed twice with 10 ml of Wash Buffer (50 mmol/L NaH_2_PO_4_, 300 mmol/L NaCl, 20 mmol/L imidazole, pH 8.0). His-PanZ protein was eluted with 4× 1 ml of Elution Buffer (50 mmol/L NaH_2_PO_4_, 300 mmol/L NaCl, 250 mmol/L imidazole, pH 8.0). Eluted His-PanZ protein was dialyzed against TBSE Buffer (50 mmol/L Tris-HCl, 150 mmol/L NaCl, 1 mmol/L EDTA, pH 7.5) and used for subsequent assays.

### Purification of PanD-FLAG

ΔPanZ cells harboring pBAD24-*PanD-flag* were incubated in 100 ml of L broth containing 50 μg/ml of ampicillin at 30°C until the OD_600_ reached 0.5, and then arabinose was added to a final concentration of 0.2% in order to express PanD-FLAG. The culture was incubated for an additional 3 h. Cells were harvested by centrifugation and resuspended in 2 ml of Lysis Buffer and incubated on ice for 30 min. Cells were disrupted by sonication and cell debris was removed by centrifugation to obtain a cleared lysate. The cleared lysate was loaded onto a column with 200 μl of Anti-FLAG M2 affinity gel (SIGMA) and washed twice with 5 ml of TBS (50 mmol/L Tris-HCl, 150 mmol/L NaCl, pH 7.5). PanD-FLAG protein was eluted with 5× 250 μl of TBS containing 100 μg/ml 3×FLAG peptide. Eluted PanD-FLAG was dialyzed against TBSE Buffer (50 mmol/L Tris-HCl, 150 mmol/L NaCl, 1 mmol/L EDTA, pH 7.5) and used for subsequent assays.

### Purification of PanD

SN227 harboring pET28Ap-PanD was cultured in L medium containing 50 μg/ml ampicillin until the OD_600_ reached 0.6, and then IPTG was added to a final concentration of 1 mmol/L. The culture was incubated for an additional 3 h. Cells were harvested by centrifugation and resuspended in Lysis buffer (50 mmol/L HEPES/KOH, 100 mmol/L potassium acetate, 1 mmol/L EDTA, 1 mg/ml lysozyme, pH 7.6). Cells were lysed by freeze-thawing three times with liquid nitrogen and a single cycle of sonication and cell debris removed by ultracentrifugation. Ammonium sulfate was added to the supernatant to 40% saturation and the precipitate was removed by ultracentrifugation. Further ammonium sulfate was added to the supernatant to 50% saturation. After ultracentrifugation, the precipitate was resuspended in K0 buffer (50 mmol/L HEPES/KOH, 1 mmol/L EDTA, pH 7.6) and dialyzed against K0 buffer for more than 3 h to remove ammonium sulfate. The dialyzed solution was loaded on a Resource Q (GE Healthcare, Uppsala, Sweden) anion-exchange column using an AKTA prime plus liquid chromatography system (GE Healthcare). Gradient elution was carried out from K0 buffer to K1000 buffer (50 mmol/L HEPES/KOH, 1 mmol/L EDTA, 1 mol/L potassium acetate, pH7.6). Each eluted fraction was analyzed by SDS-PAGE visualized using Coomassie Brilliant Blue. Protein-containing fractions were collected and dialyzed against K100 buffer (50 mmol/L Hepes/KOH, 1 mmol/L EDTA, 100 mmol/L potassium acetate, pH 7.6) containing 10% glycerol for more than 3 h. After dialysis, purified PanD protein was stored at –80°C.

### Purification of PanZ-His and PanZ(N45A)-His

SN202 harboring pBAD24-PanZ-*his* or *panZ*(N45A)-*his* was cultured in L medium containing 50 μg/ml ampicillin until the OD_600_ reached 0.6, and then l-arabinose was added to a final concentration of 0.2%. After further incubation for 3 h, cells were harvested by centrifugation and resuspended in Lysis buffer. Cells were lysed by three cycles of freeze-thawing in liquid nitrogen and a single cycle of sonication and cell debris removed by ultracentrifugation. Ammonium sulfate was added to the supernatant to a final 50% saturation. After the precipitate was removed by centrifugation, further ammonium sulfate was added to 80% saturation. After ultracentrifugation, the precipitate was resuspended in His-binding buffer (50 mmol/L HEPES/KOH, 100 mmol/L potassium acetate, 20 mM imidazole, pH 7.6) and loaded on HisTrap FF column (GE Healthcare) using the AKTA prime plus liquid chromatography system (GE Healthcare). Stepwise elution chromatography was carried out using Elution buffer (50 mmol/L HEPES/KOH, 100 mmol/L potassium acetate, 500 mmol/L imidazole, pH 7.6). Eluted proteins were analyzed by SDS-PAGE visualised using CBB. Peak fractions were collected and dialyzed against K100 buffer containing 10% glycerol. After dialysis, purified proteins were stored at –80°C.

### Assay of PanD-FLAG cleavage in vivo and in vitro

To assay in vivo PanD-FLAG cleavage, exponentially growing cells in LB broth at 37°C were harvested, and washed once with 0.85% (*w*/*v*) NaCl. The cells were lysed in 1× SDS-PAGE loading buffer and subjected to SDS polyacrylamide (20%) gel electrophoresis. After blotting of the proteins to a nitrocellulose membrane (GE Healthcare), imumunodetection using anti-FLAG M2 antibody (SIGMA) was carried out. To express His-PanZ protein in SN216 cells harboring pCA24N(-GFP)-*his-PanZ*, cells were incubated in LB broth at 37°C until the OD_600_ reached 0.2, then IPTG was added to a final concentration of 0, 1, 10 and 100 μM. The cells were incubated for an additional 60 min. SN216 cells harboring pCA24N(-GFP) were used as control.

For assay of in vitro PanD-FLAG cleavage, purified PanD-FLAG (2.5 μmol/L) was incubated with purified His-PanZ (0, 0.04, 0.16, 0.32, 0.63, 2.5, 10 and 40 μmol/L) in 50 μl of TBSE buffer (50 mmol/L Tris-HCl, 150 mmol/L NaCl, 1mmol/L EDTA, pH 7.5) for 3 h. The reaction was quenched with SDS-PAGE loading buffer and the samples were boiled for 5 min and subjected to SDS polyacrylamide (20%) gel electrophoresis. Western blotting was carried out as described above except that anti-Penta-His antibody (QIAGEN) was used for detection of His-PanZ.

### Bacterial two-hybrid assay

The bacterial two-hybrid system was applied as described by Karimova et al.(Karimova et al. 1998). All combinations of pUT18C derivatives (pUT18C, pUT18C-PanZ or pUT18C-PanD) and pKT25 derivatives (pKT25, pKT25-PanZ or pKT25-PanD) were co-transformed into the reporter strain, DHM1 on LB plates containing 50 μg/ml ampicillin and 15 μg/ml kanamycin. the positive control was the pair of pUT18C-PanD and pKT25-PanD vectors and the negative control was the empty vectors, pUT18C and pKT25. The transformed cells were spread on M9 glucose plates containing 50 μg/ml ampicillin, 15 μg/ml kanamycin and 40 μg/ml 5-bromo-4-chloro-3-indolyl-*β*-galactoside (X-gal). The plates were incubated at 30°C for 2 days.

### Phylogenetic tree of *panD*

A phylogenetic tree of PanD was generated using ClustalX (Thompson 1997) and drawn using TreeView X software (Page 1996).

### Gel filtration assay

Purified proteins were subjected to gel filtration column using the AKTA prime plus liquid chromatography system (GE Healthcare). A Superose 6 10/300 GL gel filtration column (GE Healthcare) was used at a flow rate of 0.2 ml/min of GF buffer (50 mmol/L Hepes/KOH, 150 mmol/L KCl, pH 7.6) and each 0.3 ml of eluate was fractionated from the void volume. Elution profiles of the column were calibrated using a Gel Filtration Calibration Kits (GE Healthcare) containing aprotinin (6.5 kDa), ribonuclease A (13.7 kDa), ovalbumin (44 kDa), aldolase (158 kDa), ferritin (440 kDa), thyroglobulin (669 kDa) and Blue Dextran 2000 (2000 kDa). For in vitro binding assay of PanD and PanZ-His (or PanZ(N45A)-*his*), 60 μmol/L of PanD and 15 μmol/L PanZ-His (or PanZ(N45A)-His) were mixed and incubated for 60 min at 4°C, and then 100 μl of sample was subjected to gel filtration chromatography.

## Supporting Information

**Table S1.** Oligonucleotide primers used for PCR.

**Figure S1.** Sequence alignment of *panD* from representative bacterial species. *panZ*-conserved species are indicated in a gray box. The amino acid residues are color coded as follows: Blue = Ala, Val, Leu, Ile, Met, Trp, Phe and Cys, Green = Ser, Thr, Asn and Gln, Magenta = Asp and Glu, Red = Lys and Arg, Cyan = His and Tyr, Orange = Gly, Yellow = Pro.

## References

[b1] Adams MD, Wagner LM, Graddis TJ, Landick R, Antonucci TK, Gibson AL (1990). Nucleotide sequence and genetic characterization reveal six essential genes for the LIV-I and LS transport systems of Escherichia coli. J. Biol. Chem.

[b2] Albert A, Dhanaraj V, Genschel U, Khan G, Ramjee MK, Pulido R (1998). Crystal structure of aspartate decarboxylase at 2.2 A resolution provides evidence for an ester in protein self-processing. Nat. Struct. Biol.

[b3] Baba T, Ara T, Hasegawa M, Takai Y, Okumura Y, Baba M (2006). Construction of Escherichia coli K-12 in-frame, single-gene knockout mutants: the Keio collection. Mol. Syst. Biol.

[b4] Brown GM (1959). The metabolism of pantothenic acid. J. Biol. Chem.

[b5] Chakauya E, Coxon KM, Wei M, Macdonald MV, Barsby T, Abell C (2008). Towards engineering increased pantothenate (vitamin B(5)) levels in plants. Plant Mol. Biol.

[b6] Cronan JE, (1980). β-alanine synthesis in *Escherichia coli*. J. Bacteriol.

[b7] Cronan JE, Littel KJ, Jackowski S (1982). Genetic and biochemical analyses of pantothenate biosynthesis in *Escherichia coli* and *Salmonella typhimurium*. J. Bacteriol.

[b8] Datsenko KA, Wanner BL (2000). One-step inactivation of chromosomal genes in *Escherichia coli* K-12 using PCR products. Proc. Natl. Acad. Sci. USA.

[b9] Dusch N, Puhler A, Kalinowski J (1999). Expression of the Corynebacterium glutamicum PanD gene encoding L-aspartate-α-decarboxylase leads to pantothenate overproduction in *Escherichia coli*. Appl. Environ. Microbiol.

[b10] Fouad WM, Rathinasabapathi B (2006). Expression of bacterial L-aspartate-α-decarboxylase in tobacco increases β-alanine and pantothenate levels and improves thermotolerance. Plant Mol. Biol.

[b11] Guzman LM, Belin D, Carson MJ, Beckwith J (1995). Tight regulation, modulation, and high-level expression by vectors containing the arabinose PBAD promoter. J. Bacteriol.

[b12] Jackowski S, Rock CO (1981). Regulation of coenzyme A biosynthesis. J. Bacteriol.

[b13] Joyce AR, Reed JL, White A, Edwards R, Osterman A, Baba T (2006). Experimental and computational assessment of conditionally essential genes in Escherichia coli. J. Bacteriol.

[b14] Karimova G, Pidoux J, Ullmann A, Ladant D (1998). A bacterial two-hybrid system based on a reconstituted signal transduction pathway. Proc. Natl. Acad. Sci. USA.

[b15] Kitagawa M, Ara T, Arifuzzaman M, Ioka-Nakamichi T, Inamoto E, Toyonaga H (2005). Complete set of ORF clones of *Escherichia coli* ASKA library (a complete set of *E. coli* K-12 ORF archive): unique resources for biological research. DNA Res.

[b16] Ortega MV, Cardenas A, Ubiera D (1975). PanD, a new chromosomal locus of Salmonella typhimurium for the biosynthesis of β-alanine. Mol. Gen. Genet.

[b17] van Poelje PD, Snell EE (1990). Pyruvoyl-dependent enzymes. Annu. Rev. Biochem.

[b18] Primerano DA, Burns RO (1983). Role of acetohydroxy acid isomeroreductase in biosynthesis of pantothenic acid in *Salmonella typhimurium*. J. Bacteriol.

[b19] Ramjee MK, Genschel U, Abell C, Smith AG (1997). Escherichia coli L-aspartate-α-decarboxylase: preprotein processing and observation of reaction intermediates by electrospray mass spectrometry. Biochem. J.

[b20] Schmitzberger F, Kilkenny ML, Lobley CM, Webb ME, Vinkovic M, Matak-Vinkovic D (2003). Structural constraints on protein self-processing in L-aspartate-α-decarboxylase. EMBO J.

[b21] Spry C, Kirk K, Saliba KJ (2008). Coenzyme A biosynthesis: an antimicrobial drug target. FEMS Microbiol. Rev.

[b22] Trip H, Mulder NL, Rattray FP, Lolkema JS (2011). HdcB, a novel enzyme catalysing maturation of pyruvoyl-dependent histidine decarboxylase. Mol. Microbiol.

[b23] Vallari DS, Jackowski S (1988). Biosynthesis and degradation both contribute to the regulation of coenzyme A content in *Escherichia coli*. J. Bacteriol.

[b24] Vallari DS, Rock CO (1985). Isolation and characterization of *Escherichia coli* pantothenate permease (*panF*) mutants. J. Bacteriol.

[b25] Vallari DS, Rock CO (1987). Isolation and characterization of temperature-sensitive pantothenate kinase (*coaA*) mutants of *Escherichia coli*. J. Bacteriol.

[b26] Webb ME, Smith AG, Abell C (2004). Biosynthesis of pantothenate. Nat. Prod. Rep.

[b27] Webb ME, Lobley CMC, Soliman F, Kilkenny ML, Smith AG, Blundell TL (2012). The crystal structure of Escherichia coli aspartate alpha-decarboxylase Asn72Ala: probing the role of Asn72 in pyruvoyl cofactor formation. Acta. Cryst.

[b28] Williamson JM, Brown GM (1979). Purification and properties of L-Aspartate α-decarboxylase, an enzyme that catalyzes the formation of β-alanine in *Escherichia coli*. J. Biol. Chem.

