# Supplementary material for: An activator for pyruvoyl-dependent l-aspartate α-decarboxylase is conserved in a small group of the γ-proteobacteria including Escherichia coli
Source: Microbiologyopen. 2012 Aug 14;1(3):298–310. doi: 10.1002/mbo3.34 (PMC3496974; doi:10.1002/mbo3.34)
Supplement: Supplementary file 1 [file mbo30001-0298-SD1.doc]

**Supplementary Table 1. Oligonucleotide primers used for PCR**

| Name | Oligonucleotide sequence |
| --- | --- |
| panDflagU | GCGACAATGAAATGAAACGTACCGCGAAAGCGATTCCGGTACAGGTTGCTGACTACAAGGATCATGATGG |
| panDflagL | CGCTGGAGACGATGTCGCGGCTGGTGAGTAACCAGCCGCAGGGATAACAACATATGAATATCCTCCTTAG |
| BSPD1 | AAAGCAGGCATCGCCTGCTTCGTTAACGACAGGGTAGAAAGGTAGAAGTTATGTATCGAACAATGATGAG |
| BSPD21 | CAGCCTACACCTACAAAATTGTACGGGCTG |
| BSPD31 | AATTTTGTAGGTGTAGGCTGGAGCTGCTTC |
| BSPD4 | CCTTGTAGTCCAAAATTGTACGGGCTGGTT |
| BSPD5 | TACAATTTTGGACTACAAGGATCATGATGG |
| ECpanDBADU (EcoRI)* | GGTAGAATTCGTTATGATTCGCACGATGCTGCAGGGCAAA |
| panDL (SphI)* | ATGCGCATGCAACGGATTCGCTGGAGACGA |
| panDPETU (NcoI)* | TATACCATGGTTCGCACGATGCTGCAGGGC |
| panDPETL (HindIII)* | AATTAAGCTTAACGGATTCGCTGGAGACGA |
| panZBADU (EcoRI)* | ACACGAATTCCCCATGAAGCTGACCATCATTCGATTAGAA |
| panZBADL (SphI)* | AACTGCATGCTTAACACTTCTCCCAGCCGCCCTGTTGTGC |
| panZN45AU | CGTTTTGCCGAGCGCCTGCTCGCTGCCGTG |
| panZN45AL | GCGCTCGGCAAAACGCGCGGCGTAGATACG |
| THpanZU (BamHI)* | AGAGGATCCGAAGCTGACCATCATTCGATT |
| THpanZL (EcoRI)* | GCATGAATTCGCGCATCAGGCAATATCACT |
| THpanDU (BamHI)* | AGAGGATCCGATTCGCACGATGCTGCAGGG |
| THpanDL (EcoRI)* | GCATGAATTCAAGAACGGATTCGCTGGAGA |

*, restriction enzyme recognition in primers
