# Supplementary material for: An activator for pyruvoyl-dependent l-aspartate α-decarboxylase is conserved in a small group of the γ-proteobacteria including Escherichia coli
Source: Microbiologyopen. 2012 Aug 14;1(3):298–310. doi: 10.1002/mbo3.34 (PMC3496974; doi:10.1002/mbo3.34)
Supplement: Supplementary file 2 [file mbo30001-0298-SD2.pdf]

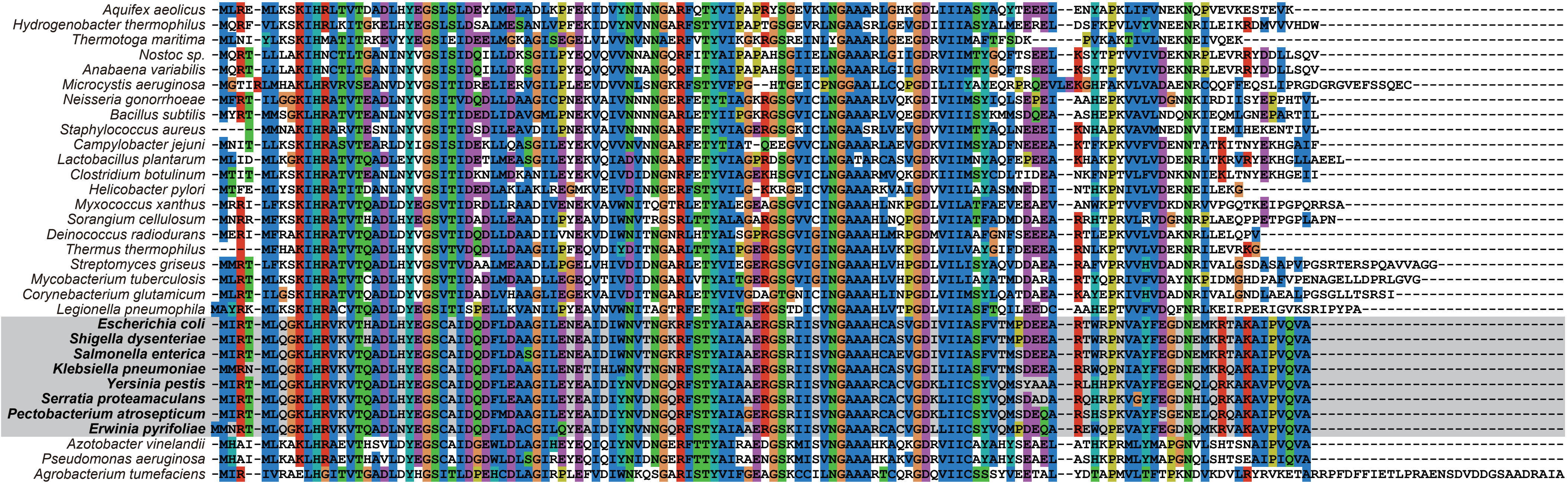

Fig. S1. **Nozaki et al.**
