# Supplementary figures and images for: An activator for pyruvoyl-dependent l-aspartate α-decarboxylase is conserved in a small group of the γ-proteobacteria including Escherichia coli
Source: Microbiologyopen. 2012 Aug 14;1(3):298–310. doi: 10.1002/mbo3.34 (PMC3496974; doi:10.1002/mbo3.34)

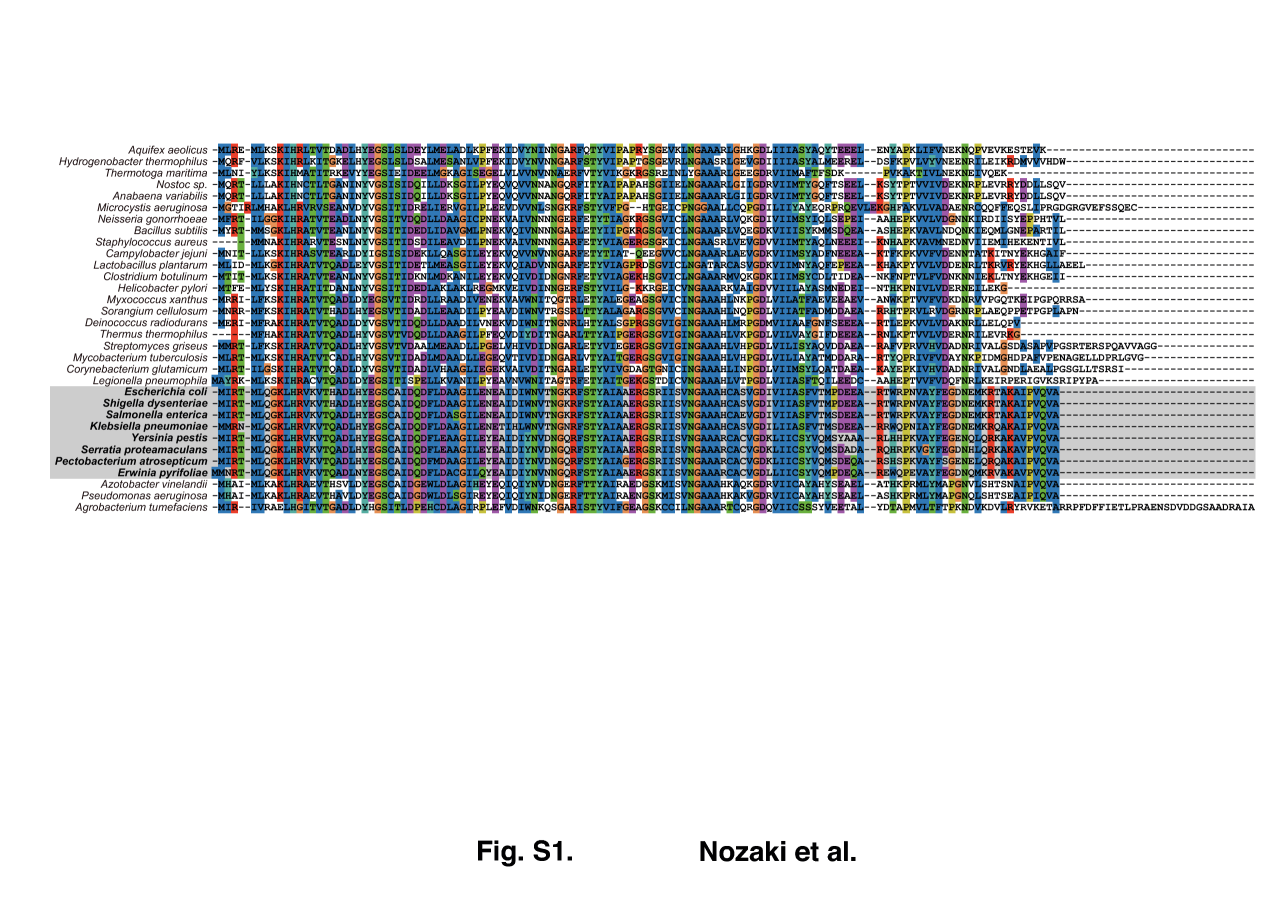

Supplement: Supplementary file 3 [file mbo30001-0298-SD3.png]
